# The Pharmacology of Acute Lung Injury in Sepsis

**DOI:** 10.1155/2011/254619

**Published:** 2011-06-28

**Authors:** Brian Michael Varisco

**Affiliations:** Division of Critical Care, Cincinnati Children's Hospital Medical Center, MLC 2005, Cincinnati, OH 45229-3039, USA

## Abstract

Acute lung injury (ALI) secondary to sepsis is one of the leading causes of death in sepsis. As such, many pharmacologic and nonpharmacologic strategies have been employed to attenuate its course. Very few of these strategies have proven beneficial. In this paper, we discuss the epidemiology and pathophysiology of ALI, commonly employed pharmacologic and nonpharmacologic treatments, and innovative therapeutic modalities that will likely be the focus of future trials.

## 1. Introduction

Acute lung injury (ALI) secondary to sepsis is the source of substantial morbidity and mortality in both adult [1, 2] and pediatric [3, 4] populations and is a major contributor to intensive care unit (ICU) costs [5]. ALI and acute respiratory distress syndrome (ARDS) are defined by well-established criteria (Table 1) [6] with sepsis and pneumonia being the two leading etiologies [2, 3].

As ARDS is associated with a risk of mortality of 26–44% in the adult population [1, 2] and 22% in the pediatric population [7], a host of therapeutic strategies have been attempted to alter the progression of ALI. While this review will focus on the pharmacology of ALI in sepsis, it will also provide brief summaries of nonpharmacologic treatment strategies. ALI will be used to refer to both ALI and ARDS unless treatments are specifically limited to patients with ARDS.

## 2. Pathophysiology of ALI in Sepsis

ALI, like sepsis, is a clinical description and common endpoint of many pathophysiologic processes and should be considered a syndrome and not a disease. In considering therapeutic strategies for ALI, clinicians attempt to treat these common processes, address underlying etiologic factors, and, when possible, tailor treatment to specific underlying pathology.

Classically, ALI has been described as progressing through three stages: exudative, proliferative, and fibrotic [8, 9]. Although different mechanisms of lung injury and severity of illnesses significantly influence the severity and duration of these stages [10], the three-stage model has remained largely intact for four decades and serves as a useful frame of reference for discussion.

**Exudative**

this initial stage of ALI encompasses the first seven days of illness and is marked by a net efflux of proteinaceous material from the intravascular to the alveolar spaces. By definition this efflux is related to increased capillary permeability (i.e., a reduced reflection coefficient) and not hydrostatic forces (i.e., an elevated left atrial pressure). The alveolar exudate reduces lung compliance and increases alveolar surface tension both by virtue of the increased viscosity of the exudate compared to air and by pulmonary surfactant neutralization [11–13]. As vascular leak occurs to varying degrees, lung compliance is heterogeneous leading to focal areas of atelectasis and the patchy bilateral infiltrate on chest X-ray classic of ALI. With positive pressure ventilation, this heterogeneous lung compliance leads to relative overdistention of more normal alveolar units and underinflation of lower compliance ones. Perfusion of inadequately ventilated lung units leads to pulmonary venous desaturation and the hypoxemia of ALI.

**Proliferative**

this second stage is a pathological fibroproliferative response to the initial injury and is classically defined as occurring during the second week. Until recently, endogenous fibroblasts were thought to mediate this response; however, emerging evidence suggests that transformation of injured epithelial cells to fibroblast-like cells (epithelial-mesenchymal transition) may play a prominent role [14]. ALI resulting from different mechanisms of injury has also been associated with the presence or absence of myofibroblasts [15, 16]. As myofibroblasts exhibit a substantially enhanced fibroproliferative response to cytokines such as transforming growth factor-*β* [17, 18], there may be a role for cytokine antagonism in these patients. Regardless of fibroblast origin or phenotype, the lung's ability to turn off the fibroproliferative response and begin tissue remodeling is a critical determinant of outcome.

**Fibrotic**

two to three weeks following the initial injury, the lung parenchyma either undergoes tissue remodeling leading resolution or the fibroproliferative response is not turned off and fibrosis results. Patients who initiate lung remodeling typically will have near-normalization of pulmonary function six months later [19]. Patients who fail to initiate lung remodeling experience progressive fibrosis which leads to worsening respiratory insufficiency and death weeks to months later. In the adult population, some patients experience initial improvement in lung function only to develop idiopathic pulmonary fibrosis months to years later. Idiopathic pulmonary fibrosis also leads to progressive respiratory insufficiency and death over the course of several months to several years [20].

## 3. Nonpharmacologic Therapies for ALI

There is no cure for ALI. Treatment is entirely supportive and aims to maintain adequate oxygenation and ventilation while minimizing secondary lung injury. The strategies by which this is done are briefly outlined below.

### 3.1. The Lung Protective Strategy

 The “Lung Protective Strategy” refers to three interventions intended to minimize secondary lung injury in patients with ALI who require mechanical ventilation. These interventions are (1) reduction of tidal volumes (volutrauma), (2) minimization of airway pressures (barotrauma), and (3) application of the minimum end expiratory pressure to prevent airway collapse (atelectrauma) [21]. A large multicenter study on ARDS showed that a 6 mL/kg tidal volume resulted in a 9% reduction in mortality compared to a 12 mL/kg volume [22]. The use of high PEEP-low fractional inspired oxygen [23], oscillatory ventilation [24, 25], or newer ventilator modes such as airway pressure release ventilation [26] have not been shown to improve mortality. There is no mortality data available on other ventilator modes such as neurally adjusted ventilator assist (NAVA) or volume support ventilation, although these modes (as well as others) have shown improvements in secondary outcomes such as oxygenation, duration of mechanical ventilation, or patient-ventilator synchrony [27].

### 3.2. Alveolar Recruitment

By virtue of the heterogeneous compliance seen in ALI, positive pressure ventilation results in overdistention of lower compliance areas of lung and underinflation of others. Maximizing alveolar recruitment should minimize these disparities. “Recruitment maneuvers” refer to several techniques that increase mean airway pressure temporarily to open closed alveoli. Prone positioning rerecruits dependent lung segments. Both recruitment maneuvers [28] and prone positioning [29, 30] have been shown to improve oxygenation but not survival.

### 3.3. Fluid Management Strategies

Adequate fluid resuscitation is a key determinant of survival in septic shock. However, fluid-overload has been associated with poorer outcomes in ALI [31]. In a recently completed randomized trial comparing liberal to restrictive fluid management after initial resuscitation, patients in the restrictive arm had significantly reduced duration of mechanical ventilation and reduced intensive care stay but no reduction in mortality [32]. Furosemide was part of the management algorithm of this trial (FACTT). To date, no trial has investigated the isolated use of furosemide in ALI, but combining albumin replacement with furosemide administration in the context of hypoproteinemia improved fluid balance and oxygenation but not mortality [33, 34]. There is an emerging consensus that after initial resuscitation, achieving a negative fluid balance is important in improving outcomes in sepsis-related ALI [35].

### 3.4. Extracorporeal Membranous Oxygenation

The use of ECMO in ALI is associated with survival in 57% of pediatric patients [36]; however, disappointing results in two early adult trials dampened enthusiasm in that population [37, 38]. A recent adult trial randomizing patients with severe ARDS to standard of care at the admitting facility versus transfer to a single ECMO center showed better outcomes in those treated with ECMO; however, no difference in outcomes was noted between the ECMO group and the conventional ventilation group at the referral center [39]. Whether the increased use of ECMO in adults seen during the H1N1 influenza pandemic [40] persists is yet to be seen.

### 3.5. Pumpless Extracorporeal Oxygenation and Carbon Dioxide Removal

In patients with adequate cardiac output, extracorporeal oxygenation and CO_2_ removal devices can reduce the ventilator work required to maintain acceptable P_a_O_2_ and P_a_CO_2_ levels. There is currently no FDA-approved device for this indication; however, several are approved for use in Canada and Europe. The devices have an advantage over traditional extracorporeal membranous oxygenation in that they require less anticoagulation and cause less hemolysis [41, 42].

## 4. Pharmacologic Therapies for ALI

The history of pharmacologic treatments for ALI is marked by many therapies that showed benefit in animal and small human trials but failed in larger human trials. Whether this is due to our inability to identify ALI subgroups or the immutability of ALI pathophysiology is a matter of conjecture.

### 4.1. Corticosteroids

The use of corticosteroids for ALI has been the subject of multiple trials [43–47] with one of them being a multicenter randomized trial [47]. The therapeutic rationale for their use is to blunt fibroproliferation. Many dosing regimens of corticosteroids have been reported, but the regimen in the largest trial [47] used a 2 mg/kg loading dose of solu-medrol, 0.5 mg/kg every 6 hours for 14 days, 0.5 mg/kg every 12 hours for 7 days, and then a taper dependent on the patient's clinical status. In the above trial, the intervention group experienced improvements in oxygenation and ventilator-free days, but no improvement in mortality. However, on subset analysis, there was a significantly increased risk of mortality in patients given solumedrol more than 14-days after ARDS onset and a trend towards improved mortality in those treated 7–14 days from ARDS onset. A meta-analysis of patients treated with corticosteroids before day 14 showed improvement in outcomes [48]. A trial from the same authors suggested benefit in starting corticosteroids within 72 hours of ARDS onset [45]. No trials have been performed to compare timing of initiation, dosing, or duration of drug administration. Particularly in the context of sepsis, early, high-dose steroid administration may slow pathogen clearance, induce myopathy, increase the risk of secondary infections, and slow wound healing. The literature supports the use of corticosteroids in ALI prior to 14 days from ALI onset. Their use should be considered in this context after a careful risk-benefit analysis.

### 4.2. *β*2-Agonists

Apart from their bronchodilator properties, *β*2-receptor signaling increases alveolar type-I cell aquaporin-5 expression and aids in alveolar fluid reabsorption [49]. *In vitro*, *ex vivo*, and preliminary human studies suggest that *β*2-agonist therapy increases alveolar fluid reabsorption and improves lung compliance [50–53]. A large randomized trial using aerosolized albuterol every 4 hours in mechanically ventilated adult patients with ARDS was terminated for futility. However, a smaller randomized trial using salbutamol infusion improved lung water and plateau airway pressures [54],leading some to speculate that inadequate drug delivery may have blunted therapeutic benefit in the larger trial.

### 4.3. Furosemide

Independent of its diuretic actions, furosemide has been shown in animal studies to improve lung function in ALI [55]. This may be secondary to the anti-inflammatory effects of furosemide, particularly its ability to reduce tumor necrosis factor-*α* levels [56].

### 4.4. Neuromuscular Blockade

Neuromuscular blockade using nondepolarizing agents is highly associated with the development of ICU myopathy, particularly in the adult population [57]. In combination with sedation and analgesia, they are generally used to facilitate ventilation and oxygenation in the most severe cases of ARDS. However, a recent single-center trial has suggested some intrinsic benefit of neuromuscular blockade in the first 48 hours of mechanical ventilation with increased ventilator-free days and reduced time in the ICU [58]. The mechanism by which this occurs is unclear.

### 4.5. Surfactant Replacement Therapy

Pulmonary surfactant improves pulmonary compliance by reducing alveolar surface tension in lower compliance alveoli thus promoting more uniform alveolar inflation. Surfactant replacement therapy is clearly beneficial in premature neonates with respiratory distress syndrome [59, 60] and also benefits neonates with lung injury secondary to infections [61]. Large randomized studies using surfactant replacement in adults have been unequivocally negative and some have tended towards harm [62–65]. Several factors may account for these differences.

1. Infants, particularly premature infants appear to be surfactant-dependent to maintaining alveolar recruitment. A normal adult has a surfactant pool size of about 22 mg of phospholipid per kg. An infant without RDS has a pool size of about 60 mg/kg and an infant with RDS has a pool size of less than 15 mg/kg [66]. Surfactant depletion is a negative predictor of extubation success in premature infants [67].
2. The developing lung does not begin alveolarization until approximately 35 weeks after conceptional age [68], and alveolarization continues through toddlerhood [69]. Pores of Kohn (alveolar) and Canals of Lambert (bronchiolar) develop at approximately one and five years of age respectively and contribute substantially to the maintenance of alveolar recruitment in the context of lung injury [70]. The lung therefore becomes able to maintain alveolar recruitment with progressively less surfactant with improved alveolar development.
3. The leak of serum proteins into the alveolar space leads to surfactant inactivation in ARDS [13], whereas the principle problem in RDS is surfactant deficiency.

Controversy exists as to whether or not surfactant replacement is helpful in the pediatric population. A single-center trial [71] and multicenter trial [72] in pediatrics showed improvements in mortality and ventilator-free days respectively with the most benefit seen in patients with primary lung infections, but both the adult and pediatric arms of a large multi-center trial using calfactant were ended early for futility. Several reviews on benefits and shortcomings of the different surfactant preparations are available [73, 74]. The clinical utility of different surfactant preparations is the source of much debate among proponents and opponents of this therapy [75].

### 4.6. Inhaled Nitric Oxide

Nitric oxide (NO) is a free-radical with a half-life of a few seconds produced by several different isoforms of nitric oxide synthase throughout the body. It is a potent pulmonary vasodilator and is currently FDA approved for use in pulmonary hypertension [76]. In conditions in which there is a large degree of pulmonary shunting (such as ALI), theoretically, inhaled NO may be used to increase pulmonary blood flow to ventilated units and improve ventilation-perfusion matching. In addition, some clinicians believe that NO may treat the secondary pulmonary hypertension seen in ALI. A recently conducted meta-analysis on the use of inhaled nitric oxide in adults and children with ALI, including fourteen randomized control studies, concluded that inhaled nitric oxide improves oxygenation but does not reduce length of ventilation, ICU stay, or mortality [77]. The general use of NO for ALI should be discouraged, although it may benefit a subset of patients with ALI.

### 4.7. Ω-3 Fatty Acids

Oxidative damage due to high fractional inspired oxygen is thought to be a substantial source of continued injury in ALI. Ω-3 fatty acids possess antioxidant properties and animal and small human trials administering supplemental Ω-3 fatty acids showed improvement in outcomes [78, 79]; however, a large randomized trial using an Ω-3 fortified enteral formula was terminated early for futility. Many confounders in the trial such as feeding intolerance, a low-mortality rate in the control group, and use of a fortified formula instead of supplements may lead to further studies in this area.

### 4.8. Liquid Ventilation

Perfluorocarbons are inert, low-surface tension liquids that have a high oxygen-carrying capacity. They may be used either to fill the lungs partially or completely. When they are used to fill the lungs completely (tidal liquid ventilation), liquid in a reservoir is oxygenated and cycled through the lung by active inspiration and exhalation. Traditional ventilation is used in partial liquid ventilation and the perflourocarbons act as a surfactant with the benefit of having high gas solubility coefficients [80]. Case series demonstrate the feasibility of using liquid ventilation in neonates [81–84], but it showed neither benefit nor harm in industry-sponsored adult trials. Further investigations are required before making recommendations regarding its use [85].

### 4.9. Activated Protein C

Multisystem organ failure (MSOF) is a common consequence of sepsis with the lung being one of the first organ systems typically involved. The pathophysiology of MSOF is complex but involves the development of diffuse microvascular thrombosis leading to local ischemia, cellular dysfunction, and cell death. Protein C is an endogenous anticoagulant that cleaves activated factors V and VIII. Levels are often pathologically low in sepsis [86]. A large multicenter randomized trial involving adult and pediatric patients with sepsis found a small but significant improvement in mortality in adults with a moderate organ dysfunction, but the pediatric arm of the trial was stopped early due to bleeding complications [87]. The use of activated protein C specifically for ALI is still in the preclinical phase [88].

### 4.10. Mesenchymal Stem Cells

MSCs are nonhematopoietic progenitor cells identified by a host of surface markers, reside in the bone marrow, and display a fibroblast-like phenotype in cell culture. MSCs were found safe in a Phase I trial of patients with acute myocardial infarction with patients receiving the MSCs having faster resolution of symptoms [89] and have shown promise in improving survival in sepsis [90] and acute kidney [91] injury among other conditions. Animal models of lung injury suggest that either intravascular [92, 93] or intratracheal [94] administration of MSCs improve lung function. Despite early concerns about engraftment [95], it now appears that although these cells traffic to the lung interstitium they do not exhibit long-term engraftment [93]. A human trial using MSCs in adults with severe ARDS is currently being developed.

## 5. Conclusions

ALI is a common complication of sepsis. Despite multiple trials, the only therapy that has demonstrated clear benefit with regards to mortality is the employment of low tidal volume ventilation strategy. Although no mortality benefit was demonstrated, a restrictive fluid strategy is well supported. Arguably, only two drugs, solumedrol and furosemide, have shown therapeutic benefit. Among the other therapies listed, their general use cannot be advocated but may be beneficial to select patients. We do not yet have the ability to phenotype ALI in a clinically meaningful way. As ALI is common in ICUs and associated with significant morbidity, mortality, and cost, investigators will continue to explore new pharmacologic and nonpharmacologic therapies despite a long history of disappointments.

## Figures and Tables

**Table 1:** Diagnostic Criteria for ALI and ARDS [6].

ALI Criteria	ARDS Criteria
Acute Onset	Acute Onset
P_a_O_2_/FiO_2_ ≤ 300 mmHg	P_a_O_2_/FiO_2_ ≤ 200 mmHg
Chest Radiograph: Bilateral infiltrates	Chest Radiograph: Bilateral infiltrates
No evidence of left atrial hypertension	No evidence of left atrial hypertension
